# Engagement in mHealth-Prompted Self-Measured Blood Pressure Monitoring Among Participants Recruited From a Safety-Net Emergency Department: Secondary Analysis of the Reach Out Trial

**DOI:** 10.2196/54946

**Published:** 2024-06-12

**Authors:** Lesli E Skolarus, Chun Chieh Lin, Sonali Mishra, William Meurer, Mackenzie Dinh, Candace Whitfield, Ran Bi, Devin Brown, Rockefeller Oteng, Lorraine R Buis, Kelley Kidwell

**Affiliations:** 1 Department of Neurology, Stroke and Vascular Neurology, Northwestern University, Chicago, IL, United States; 2 Department of Neurology, The Ohio State University, Columbus, OH, United States; 3 Department of Internal Medicine-Cardiology, University of Michigan, Ann Arbor, MI, United States; 4 Department of Emergency Medicine, University of Michigan, Ann Arbor, MI, United States; 5 Department of Neurology, University of Michigan, Ann Arbor, MI, United States; 6 Department of Family Medicine, University of Michigan, Ann Arbor, MI, United States; 7 Department of Biostatistics, University of Michigan, Ann Arbor, MI, United States

**Keywords:** hypertension, self-measured blood pressure, mobile health, blood pressure, emergency, blood pressure monitoring, risk factor, cardiovascular, cardiovascular disease, utilization, feedback, care, systolic blood pressure, emergency department, mHealth, health disparities, engagement

## Abstract

**Background:**

Hypertension, a key modifiable risk factor for cardiovascular disease, is more prevalent among Black and low-income individuals. To address this health disparity, leveraging safety-net emergency departments for scalable mobile health (mHealth) interventions, specifically using text messaging for self-measured blood pressure (SMBP) monitoring, presents a promising strategy. This study investigates patterns of engagement, associated factors, and the impact of engagement on lowering blood pressure (BP) in an underserved population.

**Objective:**

We aimed to identify patterns of engagement with prompted SMBP monitoring with feedback, factors associated with engagement, and the association of engagement with lowered BP.

**Methods:**

This is a secondary analysis of data from Reach Out, an mHealth, factorial trial among 488 hypertensive patients recruited from a safety-net emergency department in Flint, Michigan. Reach Out participants were randomized to weekly or daily text message prompts to measure their BP and text in their responses. Engagement was defined as a BP response to the prompt. The k-means clustering algorithm and visualization were used to determine the pattern of SMBP engagement by SMBP prompt frequency—weekly or daily. BP was remotely measured at 12 months. For each prompt frequency group, logistic regression models were used to assess the univariate association of demographics, access to care, and comorbidities with high engagement. We then used linear mixed-effects models to explore the association between engagement and systolic BP at 12 months, estimated using average marginal effects.

**Results:**

For both SMBP prompt groups, the optimal number of engagement clusters was 2, which we defined as high and low engagement. Of the 241 weekly participants, 189 (78.4%) were low (response rate: mean 20%, SD 23.4) engagers, and 52 (21.6%) were high (response rate: mean 86%, SD 14.7) engagers. Of the 247 daily participants, 221 (89.5%) were low engagers (response rate: mean 9%, SD 12.2), and 26 (10.5%) were high (response rate: mean 67%, SD 8.7) engagers. Among weekly participants, those who were older (>65 years of age), attended some college (vs no college), married or lived with someone, had Medicare (vs Medicaid), were under the care of a primary care doctor, and took antihypertensive medication in the last 6 months had higher odds of high engagement. Participants who lacked transportation to appointments had lower odds of high engagement. In both prompt frequency groups, participants who were high engagers had a greater decline in BP compared to low engagers.

**Conclusions:**

Participants randomized to weekly SMBP monitoring prompts responded more frequently overall and were more likely to be classed as high engagers compared to participants who received daily prompts. High engagement was associated with a larger decrease in BP. New strategies to encourage engagement are needed for participants with lower access to care.

## Introduction

Hypertension is the most important modifiable risk factor for cardiovascular disease and is more prevalent among Black and low-income people [1-3]. Given the ubiquity and inequities of hypertension, scalable approaches are needed to identify and treat Americans with hypertension.

One scalable approach may be by partnering with safety-net emergency departments (EDs)—hospitals where over 25% of patients are Medicaid recipients—to reach a medically underserved population to initiate mobile health (mHealth) interventions. There are over 136 million ED visits annually [4], and Black Americans and those with low incomes frequently use the ED [5].

mHealth is readily scalable as nearly all American adults (96%) have a mobile phone and over 80% use it for text messaging [6]. Text messaging is a leading form of communication partly because of its cost, ease, and low technical threshold. mHealth hypertension interventions have primarily targeted medication adherence, self-management (including diet and physical activity), and self-measured blood pressure (SMBP) monitoring. SMBP monitoring involves the regular measurement of BP by a patient outside the clinical setting. The outcomes of these mHealth hypertension interventions have had mixed results [7-10]. SMBP reduces BP, in part by promoting the habit of medication adherence and a healthy lifestyle, but little is known about SMBP in the Black and low-income population [11-15]. Additionally, more work is needed to understand engagement with mHealth text messaging interventions and how engagement relates to clinical outcomes.

“Engagement” has been conceptualized differently across fields and studies, but generally refers to users’ use of and experience with the system [16]. Although engagement with an intervention is distinct from the performance of health behavior, as users may abandon the use of an intervention while continuing to perform the target behavior [17,18], engagement is associated with intervention efficacy in multiple health domains [19]. Engagement with an intervention may describe interaction with either push- or pull-based interventions [20]. The former describes a user responding to an intervention, for instance, by responding to a text message; the second describes the user calling on the intervention in response to their own needs, for instance, by opening a dashboard to review their past BP measurements. Although push interventions have the potential to increase users’ performance of critical health behaviors, such as SMBP, they run the risk of burdening or annoying users, leading to disengagement [20]. Despite work to categorize and evaluate the efficacy of various engagement strategies for mHealth interventions [21], dropout rates remain high [22]. More work is needed to understand how users interact with mHealth interventions, their target health behaviors, and what factors contribute to engagement or disengagement with the intervention.

In this study, we examine engagement with a push intervention in which participants were asked to text SMBP measurements to the research team in response to a text prompt. We identify patterns of engagement with SMBP monitoring, factors associated with engagement, and the association of engagement with lowered BP.

## Methods

### Ethical Considerations

This study was approved by the University of Michigan Institutional Review Board (HUM00138470) and the ED site Institutional Review Board (1199877). All participants provided written informed consent. The original informed consent allowed for deidentified use in additional analyses and research studies. Participants of the Reach Out trial were given an automated BP cuff, US $20 at enrollment, US $25 after the completion of a 6-month follow-up visit, and US $30 after the completion of a 12-month follow-up visit. If needed, transportation was provided to follow-up visit(s).

### Design

This is a nonprespecified secondary analysis of data from the Reach Out Trial. Reach Out was a randomized, controlled, 2×2×2 factorial design mHealth clinical trial to reduce BP among hypertensive safety-net ED patients seeking care for conditions likely to be discharged from the ED (identifier NCT03422718 on ClinicalTrials.gov) [23]. Enrollment occurred from March 2019 to March 2020. Participants were randomized to (1) prompted SMBP monitoring (daily vs weekly) with feedback, (2) tailored healthy behavior text messaging (daily vs none), and (3) facilitated primary care provider (PCP) scheduling and transportation (yes vs no) for 12 months. There were minimal differences across the intervention arms [24]. Among participants, BP declined over the 12-month intervention period. There was no difference in change in systolic BP among the 3 mHealth components [24].

### Prompted SMBP Monitoring With Feedback

Participants were randomized to receive daily or weekly automated text prompts to take their BP and text the results to the study team. All participants were given a BP cuff at the time of enrollment. Sample prompts included the following: “This is your reminder to take your BP. REPLY with your BP to Reach Out!” Participants randomized to weekly prompted SMBP monitoring were sent up to 2 follow-up reminders over a 24-hour period if they remained unresponsive. Participants randomized to daily prompted SMBP monitoring were not sent follow-up reminders. All participants received an automated confirmation text for each BP texted to the study team. Each week, all participants received a tailored feedback message comparing the participant’s recent BP to goal BP, along with general encouragement. For example, if a participant reported an SMBP with systolic BP higher than the threshold but diastolic BP lower than the threshold, the following text was sent: “Your BP is 150/75. Your top number is above the normal range, but your bottom number is normal. Meds, eating healthy, and exercise can lower that top number!” Participants also received a monthly text that contained a graph of their self-reported BPs, with tailored interpretation. In this context, engagement was defined as a BP response to an SMBP prompt.

### Covariates and BP

Covariates were chosen based on prior research suggesting an association with engagement in SMBP monitoring [13]. Age, race (self-reported Black vs non-Black), sex, education (no college vs any college), insurance type (eg, Medicaid, Medicare, private, uninsured, multiple, or other types of insurance), relationship status (living with someone or married vs living alone), and employment status were self-reported. We also queried access to hypertension care, including the presence of a PCP [25], diagnosis of hypertension, prior hypertension medication in the last 6 months, and inability to attend medical appointments due to lack of transportation (yes vs no). Finally, we queried medical comorbidities (eg, stroke, congestive heart failure, myocardial infarction, and kidney disease) [25].

Baseline BP measurements were the mean of the median of the remote systolic BPs received during the 3-week eligibility phase. All BP outcomes were intended to be measured in person. However, outcomes were assessed remotely for some participants at 6 months and for all participants at 12 months due to COVID-19 research restrictions. In-person BP assessments were conducted by trained research team members using an OMRON 7 Series Upper Arm BP Monitor and following standard procedures [26]. If performed remotely, participants were asked to provide 3 BP measurements, each 1 minute apart. Participants communicated these to the study team through phone calls or text messages. Participants were also asked to send a picture of their BP cuff on their arms to confirm the correct orientation.

### Analysis

We used the k-means clustering algorithm and visualization to determine the pattern of SMBP monitoring engagement by SMBP prompt frequency. For both prompted SMBP frequency groups (weekly and daily), the optimal number of clusters of engagement via the elbow method was 2, which we defined as high and low engagement (Multimedia Appendices 1-4) [27]. For each daily and weekly prompted SMBP frequency group, we used descriptive statistics to describe engagement type and assessed the univariate association of demographics, access to care, and comorbidities with high engagement using logistic regression models. Finally, we sought to determine whether engagement was associated with a difference in systolic BP at 12 months using a linear mixed-effects model with 12-month systolic BP as the outcome variable and baseline BP, engagement type (high vs low), time measured in days from randomization to 1 year, and the interaction of time and engagement type as fixed effects, with a random participant effect. Systolic BP was estimated using average marginal effects from the fully adjusted model. K-mode clustering was performed using Python (version 3.11.2; Python Software Foundation), and all other analyses were conducted using SAS (version 9.4; SAS Institute).

## Results

### Participants

A total of 488 participants were randomized into the intervention; 241 (49.4%) were randomized to weekly monitoring and 247 (50.6%) were randomized to daily monitoring. Within this safety-net ED, of the 241 randomized participants, 117 (48.6%) used Medicaid. Demographics, access to care, and comorbidities for each prompted SMBP frequency group are included in Tables1  2.

**Table 1.** Association of engagement among participants who received weekly blood pressure prompts.

Characteristics		Weekly total, n (%)	Low engager, n (%)	High engager, n (%)	Univariate analysis in predicting high engagers	
					Odds ratio (95% CI)	*P* value
Total		241 (100)	189 (78.4)	52 (21.6)	—^a^	—
**Demographics**						
	Older than 65 years	19 (7.9)	8 (4.2)	11 (21.2)	6.1 (2.3‐16.0)	<.001
	Women	148 (61.4)	117 (61.9)	31 (59.6)	0.9 (0.5‐1.7)	.76
	Non-Hispanic Black people	132 (54.8)	105 (55.6)	27 (51.9)	0.9 (0.5‐1.6)	.64
	Married or living with someone	60 (24.9)	40 (21.2)	20 (38.5)	2.3 (1.2‐4.5)	.01
	Not employed	123 (51.0)	94 (49.7)	29 (55.8)	1.3 (0.7‐2.4)	.44
**Education**						
	Some high school education, high school graduate, or trade school	118 (49.0)	102 (54.0)	16 (30.8)	1	—
	Some college education or college graduate	123 (54.8)	87 (46.0)	36 (69.2)	2.6 (1.4‐5.1)	.004
**Access to care**						
	Under the care of a primary care doctor	195 (80.9)	146 (77.3)	49 (94.2)	4.8 (1.4‐16.2)	.01
	Previous diagnosis of hypertension	190 (78.8)	146 (77.3)	44 (84.6)	1.6 (0.7‐3.7)	.25
	Prior medication for hypertension in the last 6 months	136 (56.4)	96 (50.8)	40 (76.9)	3.2 (1.6‐6.5)	.001
	Lack of transportation for medical appointments	49 (20.3)	45 (23.8)	4 (7.7)	0.3 (0.1‐0.8)	.02
**Health insurance**						
	Medicaid	117 (48.6)	101 (53.4)	16 (30.8)	1	—
	Private	50 (20.8)	38 (20.1)	12 (23.1)	2.0 (0.9‐4.6)	.11
	Medicare	22 (9.1)	10 (5.3)	12 (23.1)	7.6 (2.8‐20.4)	<.001
	Other insurance	5 (2.1)	4 (2.1)	1 (1.9)	1.6 (0.2‐15.0)	.69
	Uninsured	19 (7.9)	15 (7.9)	4 (7.7)	1.7 (0.5‐5.7)	.40
	Multiple insurances	28 (11.6)	21 (11.1)	7 (13.5)	2.1 (0.8‐5.8)	.15
**Comorbidities**						
	Stroke or transient ischemic attack	17 (7.1)	13 (6.9)	4 (7.7)	1.1 (0.4‐3.6)	.84
	Congestive heart failure	12 (5.0)	7 (3.7)	5 (9.6)	2.8 (0.8‐9.1)	.09
	Myocardial infarction	13 (5.4)	8 (4.2)	5 (9.6)	2.4 (0.8‐7.7)	.14
	Kidney disease	16 (6.6)	10 (5.3)	6 (11.5)	2.3 (0.8‐6.8)	.12
**Intervention components**						
	Healthy behavior texts	121 (50.2)	100 (52.9)	21 (40.4)	0.6 (0.3‐1.1)	.11
	Primary care provider–facilitated scheduling and transportation	119 (49.4)	94 (49.7)	25 (48.1)	0.9 (0.5‐1.7)	.83

a Not applicable.

**Table 2.** Association of engagement among participants who received daily blood pressure prompts.

Characteristics		Daily total, n (%)	Low engager, n (%)	High engager, n (%)	Univariate analysis in predicting high engagers	
					Odds ratio (95% CI)	*P* value
Total		247 (100)	221 (89.5)	26 (10.5)	—^a^	—
**Demographics**						
	Older than 65 years	18 (7.3)	15 (6.8)	3 (11.5)	1.8 (0.5‐6.7)	.38
	Women	151 (61.1)	132 (59.7)	19 (73.1)	1.8 (0.7‐4.5)	.19
	Non-Hispanic Black people	130 (52.6)	120 (54.3)	10 (38.5)	0.5 (0.2‐1.2)	.13
	Married or living with someone	65 (26.3)	54 (24.4)	11 (42.3)	2.3 (1.0‐5.2)	.06
	Not employed	119 (48.2)	105 (47.5)	14 (53.9)	1.3 (0.6‐2.9)	.54
**Education**						
	Some high school education, high school graduate, or trade school	122 (49.4)	113 (51.1)	9 (34.6)	1	—
	Some college education or college graduate	125 (50.6)	108 (48.9)	17 (65.4)	2.0 (0.9‐4.6)	.12
**Access to care**						
	Under the care of a primary care doctor	185 (74.9)	163 (73.8)	22 (84.6)	2.0 (0.7‐5.9)	.23
	Previous diagnosis of hypertension	195 (79.0)	173 (78.3)	22 (84.6)	1.5 (0.5‐4.6)	.46
	Prior medication for hypertension in the last 6 months	137 (55.5)	119 (53.9)	18 (69.2)	1.9 (0.8‐4.6)	.14
	Lack of transportation for medical appointments	52 (21.1)	49 (22.2)	3 (11.5)	0.5 (0.1‐1.6)	.22
**Health insurance**						
	Medicaid	127 (51.4)	119 (53.9)	8 (30.8)	1	—
	Private	48 (19.4)	41 (18.6)	7 (26.9)	2.5 (0.9‐7.4)	.09
	Medicare	23 (9.3)	21 (9.5)	2 (7.7)	1.4 (0.3‐7.1)	.67
	Other insurance	3 (1.2)	3 (1.4)	0 (0)	0.0 (<0->1000)	.99
	Uninsured	29 (11.8)	25 (11.3)	4 (15.4)	2.4 (0.7‐8.5)	.18
	Multiple insurances	17 (6.9)	12 (5.4)	5 (19.2)	6.2 (1.8‐22.0)	.005
**Comorbidities**						
	Stroke or transient ischemic attack	19 (7.7)	16 (7.2)	3 (11.5)	1.7 (0.5‐6.2)	.40
	Congestive heart failure	11 (4.5)	11 (5.0)	0 (0)	<0.01 (<0.01->999.99)	.97
	Myocardial infarction	18 (7.3)	15 (6.8)	3 (11.5)	1.8 (0.5‐6.7)	.38
	Kidney disease	9 (3.6)	9 (4.1)	0 (0)	<0.01 (<0.01->999.99)	.98
**Intervention components**						
	Healthy behavior texts	120 (48.6)	108 (48.9)	12 (46.2)	0.9 (0.4‐2.0)	.79
	Primary care provider–facilitated scheduling and transportation	123 (49.8)	109 (49.3)	14 (53.9)	1.2 (0.5‐2.7)	.66

a Not applicable.

### Weekly SMBP

**Figure 1.**
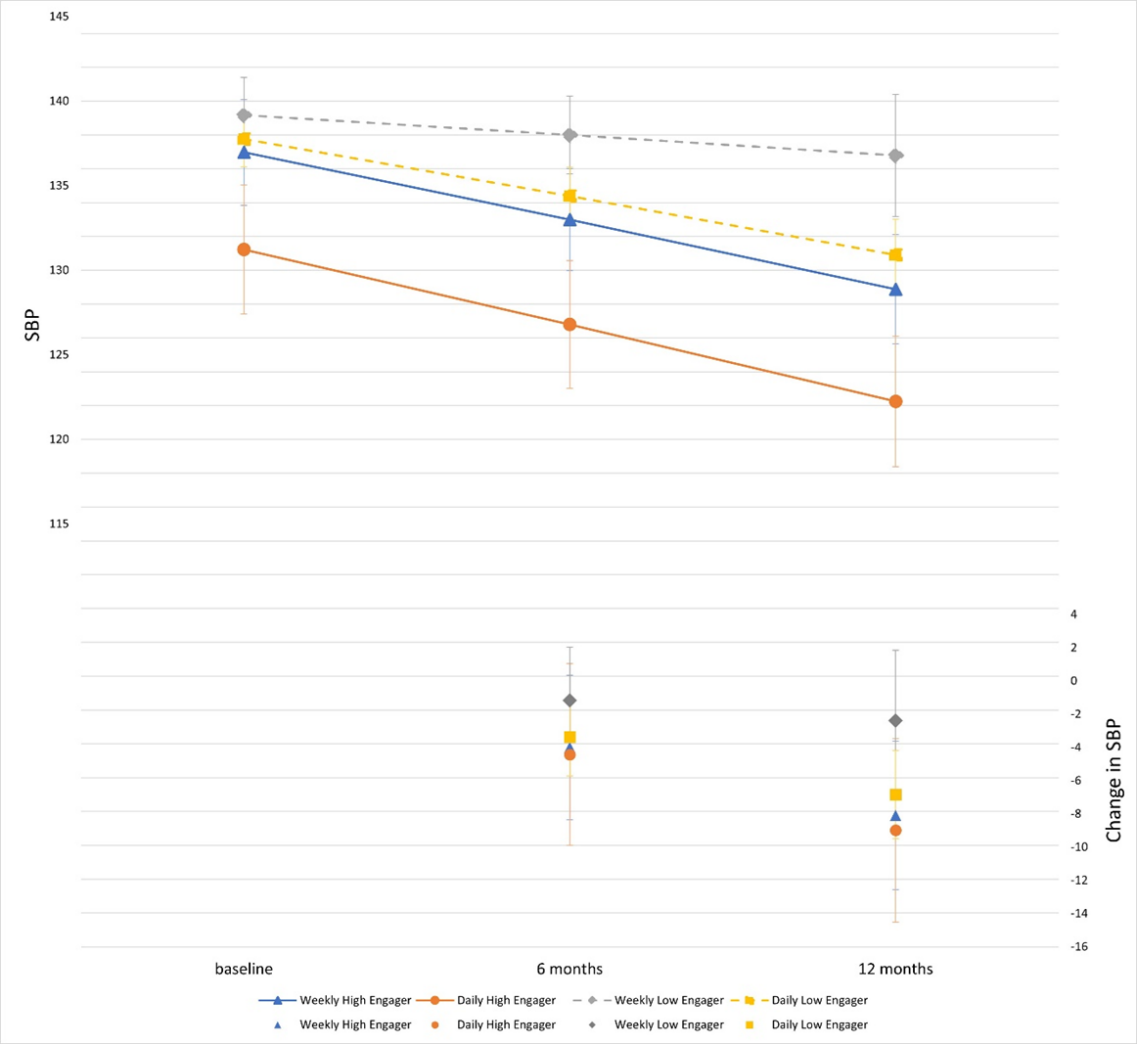
Estimated blood pressure (BP) and change in BP by engagement and time among participants who received BP prompts. SBP: systolic blood pressure.

Of the 241 participants randomized to weekly SMBP prompts, 189 (78.4%) were classed as low engagers, and 52 (21.6%) were classed as high engagers (Table 1). Low engagers had a mean response rate of 20% (SD 23.4), while high engagers had a mean response rate of 86% (SD 8.7). Participants who were low engagers decreased their engagement early in the trial (Multimedia Appendix 3). Participants older than 65 years (odds ratio [OR] 6.1, 95% CI 2.3‐16.0; *P*<.001), married or living with someone compared to living alone (OR 2.3, 95% CI 1.2‐4.5; *P*=.01), and those who attended some college (OR 2.6, 95% CI 1.4‐5.1; *P*=.004) compared to those without college attendance were more likely to be high engagers. Participants with more access to care, including participants with Medicare (OR 7.6, 95% CI 2.8‐20.4; *P*<.001) compared to participants with Medicaid, those with the care of a primary care doctor (OR 4.8, 95% CI 1.4‐16.2; *P*=.01), and those taking antihypertensive medication in the last 6 months (OR 3.2, 95% CI 1.6‐6.5; *P*=.001) had higher odds of high engagement. Participants who lacked transportation to appointments (OR 0.3, 95% CI 0.1‐0.8; *P*=.02) had lower odds of high engagement. There was no association between engagement with PCP facilitation and healthy behavior text messaging. Participants who were high engagers had a greater decline in BP (−8.1 mm Hg, 95% CI −12.6 to −3.6), compared to low engagers (−2.4 mm Hg, 95% CI −6.6 to 1.9), and lower BP at 12 months (high engagers: 128.9 mm Hg, 95% CI 125.7‐132.1 vs low engagers: 136.8 mm Hg, 95% CI 133.2‐140.4; Figure 1 and Table 3 ).

**Table 3.** Estimated blood pressure by engagement and time among participants who received blood pressure prompts.

SBP^a^ prompts	High engager					Low engager				
	BL^b^SBP (mm Hg; 95% CI)	6-monthSBP (mm Hg; 95% CI)	Change from BL to 6-monthSBP (95% CI)	12-monthSBP (mm Hg; 95% CI)	Change from BL to 12-monthSBP(95% CI)	BLSBP (mm Hg; 95% CI)	6-monthSBP (mm Hg; 95% CI)	Change from BL to 6-monthSBP(95% CI)	12-monthSBP (mm Hg; 95% CI)	Change from BL to 12-monthSBP(95% CI)
Weekly	137.0(133.8 to 140.1)	133.0(130.0 to 136.0)	−4.0(−8.3 to 0.4)	128.9(125.7 to 132.1)	−8.1(−12.6 to −3.6)	139.2(136.9 to 141.4)	138.0(135.7 to 140.3)	−1.2(−4.4 to 2.02)	136.8(133.2 to 140.4)	−2.4(−6.6 to 1.9)
Daily	131.2(127.4 to 135.1)	126.8(123.0 to 130.6)	−4.4(−9.9 to 1.04)	122.2(118.4 to 126.1)	−9.0(−14.5 to −3.5)	137.8(136.1 to 139.4)	134.4(132.7 to 136.1)	−3.4(−5.7 to −1.03)	130.9(128.8 to 133.0)	−6.8(−9.5 to −4.2)

a SBP: systolic blood pressure.

b BL: baseline.

### Daily SMBP

Of the 247 participants randomized to daily SMBP prompts, 221 (89.5%) were classed as low engagers and 26 (10.5%) as high engagers (Table 2). Low engagers had a response rate of 9% (SD 12.2), while high engagers had a mean response rate of 67% (SD 8.7). Participants who were low engagers decreased their engagement early in the trial (Multimedia Appendix 4). Participants who were married or living with someone had a trend toward high engagement (OR 2.3 95% CI 1.0‐5.2; *P*=.06), and participants with multiple insurance types were more likely to have high engagement (OR 6.2, 95% CI 1.8‐22.0; *P*=.005) compared to Medicaid alone. Participants who were high engagers had a greater decline in BP (−9.0 mm Hg, 95% CI −14.5 to −3.5) compared to low engagers (−6.8 mm Hg, 95% CI −9.5 to −4.2) and lower BP at 12 months (high engagers: 122.2 mm Hg, 95% CI 118.4‐12.1 vs low engagers 130.9 mm Hg, 95% CI 128.8‐133.0; Figure 1 and Table 3).

## Discussion

In this secondary analysis of a mHealth clinical trial among participants recruited from a safety-net ED, we identified 2 distinct patterns of SMBP engagement.

### High and Low Engagement: Interpretation and Implications

In both daily and weekly reminder conditions, participants could be clustered into high and low engagers. A larger proportion of participants randomized to weekly SMBP monitoring prompts were high engagers and had greater engagement overall compared to participants who received daily prompts. All engagement groups had a decline in BP, even low engagers in the daily condition who exhibited a very low response rate.

In both daily and weekly conditions, many of the factors associated with high engagement were factors also associated with better health outcomes in general: high engagers generally had attended college, had treated their hypertension with an antihypertensive medication in the last 6 months, had Medicare, and had more access to care (ie, they had reported a PCP at enrollment or reported having transportation to medical appointments). Perhaps due to these factors, high engagers had slightly lower baseline systolic BP than low engagers in both conditions (Table 3). High engagers also exhibited a greater decrease in BP than low engagers. This may be related to their baseline characteristics, which could promote a habit of medication adherence and a practice of a healthy lifestyle (through SMBP) [11-15]. Another possible explanation for this finding, which would require further study, is that high engagers learned more about what impacted their BP from frequent monitoring. In this case, researchers should explore strategies for promoting engagement to turn low engagers into high engagers.

We found that engagement levels declined quickly and did not recover, suggesting re-engagement attempts should begin early on. Re-engagement strategies could take the form of direct outreach from the study team. However, for the sake of scalability, researchers should also explore techniques for re-engaging participants through the intervention itself. Although there is not yet a good evidence base for re-engagement strategies for mHealth interventions, existing research suggests that when users re-engage, it is for the same health motivations that brought them to the system in the first place [28]. Work examining the adoption and re-adoption of wearables has also found that the visual appeal of new devices is also associated with re-engagement [29]. These findings suggest that reminding users of their health goals and prompting them to monitor their BP could be an effective re-engagement technique. Although researchers have little control over the general visual appeal of text messages, future interventions could explore whether varying the form of reminders to include more graphical elements, for instance, by sending images as text messages, could promote engagement or re-engagement.

Another possible explanation of our findings is that high engagers were more likely to engage in SMBP because they were better able to act on their BP. For instance, high engagers may have been able to talk to their PCP about their recorded measurements [30]. By contrast, low engagers, who overall had less access to care, may have been less able to consult clinicians about what additional management was needed, leading to discouragement and disengagement. This interpretation suggests that SMBP alone is unable to mitigate the impact of decreased access to care. Additional strategies, such as connecting participants to primary care—which was initiated in the Reach Out trial but ended due to COVID-19, the addition of community-health worker support, or activating social networks may be needed.

### Prompt Frequency, Engagement, and BP

Participants in the weekly condition exhibited higher engagement, and higher engagement was associated with a greater decrease in BP. The lower engagement observed in the daily condition could be due to habituation, as observed in other mHealth contexts [17,31]—users may simply have gotten used to the notifications and begun ignoring them. Lower engagement could signal frustration or dissatisfaction with the intervention, impacting real-world use. Other studies had similar findings—higher engagement was linked to improved outcomes [32,33]. More work is needed to evaluate the reasons for higher engagement. To get better insight into the effect of each message, future work could analyze not just the response rate but also the rate at which messages are read and the rate at which particular behaviors associated with hypertension management, such as medication taking, are performed. Such granular data could enable researchers to get more insight into how different doses of the intervention function. Future works could also focus on the efficacy of prompt frequency alongside habituation to better inform how to optimize engagement.

### Limitations and Conclusions

Our study has limitations. This was a nonprespecified secondary analysis of clinical trial data. Reach Out was a single-center trial; thus, results may not be generalizable to other safety-net ED populations, particularly those with non–English-speaking populations. Finally, we cannot exclude that a small subset of participants completed SMBP monitoring but did not text in their BPs, and as a result, we may be overestimating the proportion of low engagers. If high engagers were misclassified as low engagers, this should bias our findings toward the null.

In conclusion, about 16% of safety-net ED participants were highly engaged in prompted SMBP monitoring with feedback. Participants in the weekly condition overall exhibited higher engagement compared to daily prompts. BP decreased among all participants, but those with higher engagement had a greater decline in BP overall, supporting weekly rather than daily BP prompts. New strategies to encourage engagement are needed for participants who were not taking antihypertensive medication and had lower access to care.

## Supplementary material

10.2196/54946 Multimedia Appendix 1 Weekly self-measured blood pressure engagement of optimal K.

10.2196/54946 Multimedia Appendix 2 Daily self-measured blood pressure engagement of optimal K.

10.2196/54946 Multimedia Appendix 3 Frequency of self-measured blood pressure (SMBP) response to weekly SMBP prompts clustered by engagement.

10.2196/54946 Multimedia Appendix 4 Frequency of self-measured blood pressure (SMBP) response to daily SMBP prompts clustered by engagement.

## Abbreviations

BP: blood pressure
ED: emergency department
mHealth: mobile health
OR: odds ratio
PCP: primary care provider
SMBP: self-measured blood pressure
