# Supplementary figures and images for: Engagement in mHealth-Prompted Self-Measured Blood Pressure Monitoring Among Participants Recruited From a Safety-Net Emergency Department: Secondary Analysis of the Reach Out Trial
Source: JMIR Mhealth Uhealth. 2024 Jun 12;12:e54946. doi: 10.2196/54946 (PMC11186514; doi:10.2196/54946)

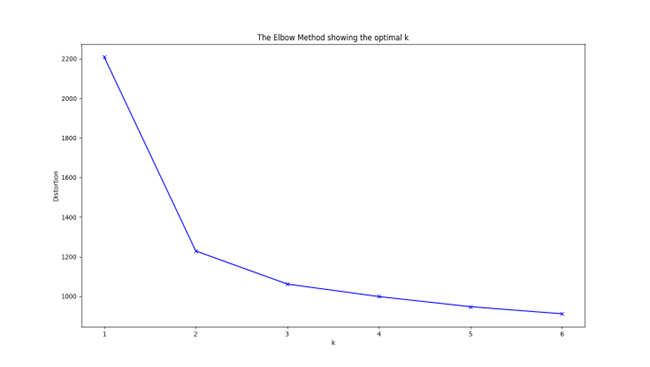

Supplement: Multimedia Appendix 1 [file mhealth-v12-e54946-s001.png]

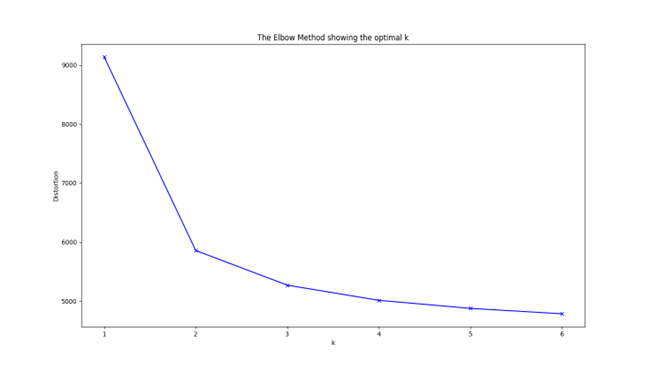

Supplement: Multimedia Appendix 2 [file mhealth-v12-e54946-s002.png]

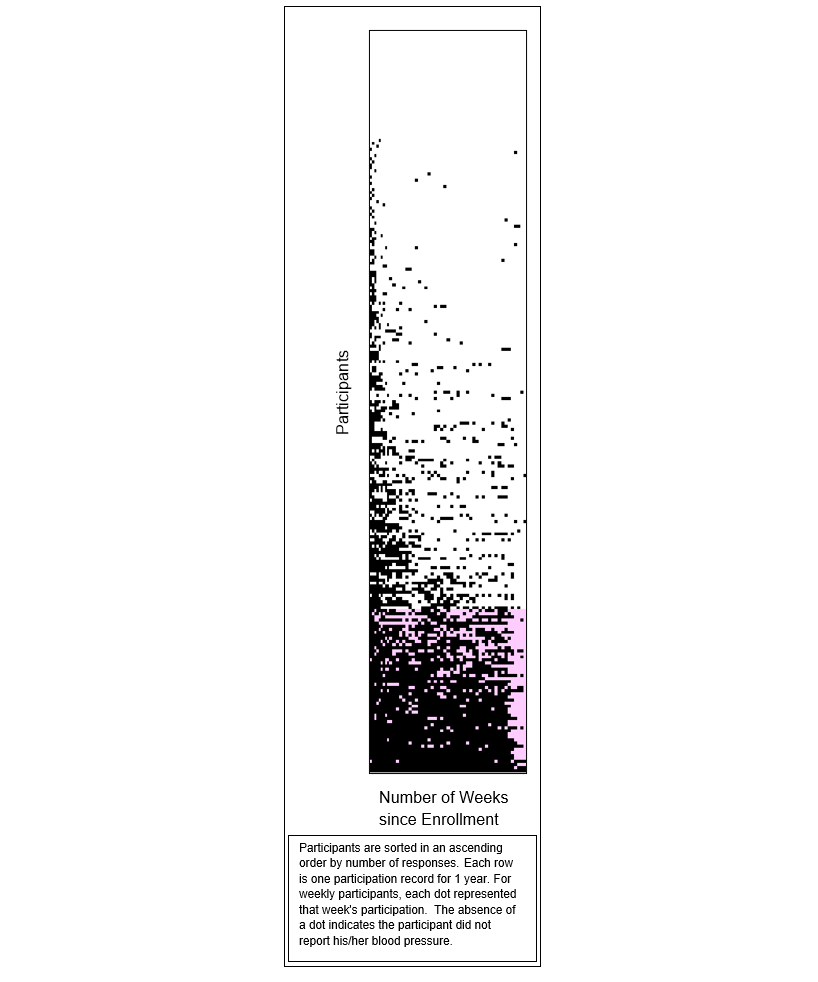

Supplement: Multimedia Appendix 3 [file mhealth-v12-e54946-s003.png]

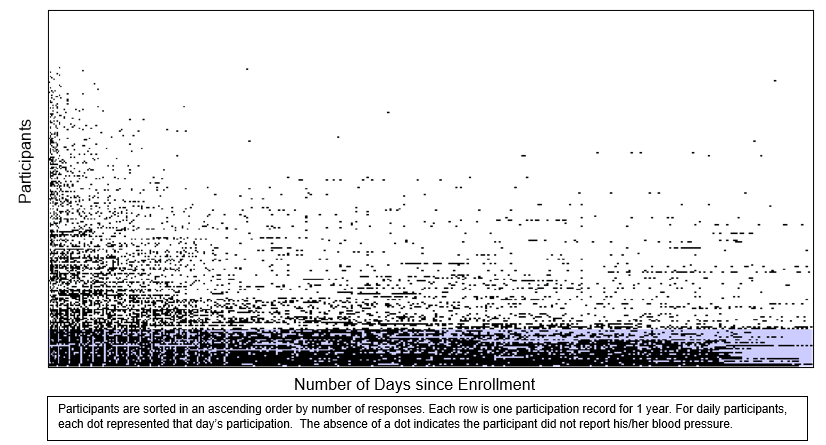

Supplement: Multimedia Appendix 4 [file mhealth-v12-e54946-s004.png]
